# Factors associated with the duration of breastfeeding in mothers of babies cared for in a kangaroo family program*

**DOI:** 10.17533/udea.iee.v40n3e08

**Published:** 2023-02-10

**Authors:** Isabel-Cristina Giraldo-Marín, Natalia Andrea Henao Murillo, María Camila Londoño Rodríguez, Manuela Aguirre Torres, Gabriel Jaime López Palacio

**Affiliations:** 1 Nurse, Master. Teacher. Catholic University of the East, Rionegro, Colombia. Email: isabelgir10@gmail.com Catholic University of the East Rionegro Colombia isabelgir10@gmail.com; 2 Nurse, Master. Teacher. Catholic University of the East, Rionegro Email: nathyh75@gmail.com Catholic University of the East Rionegro nathyh75@gmail.com; 3 Nurse. Neonatal Intensive Care Unit in SOMER Clinic, Rionegro, Colombia. Email: macalodri12@gmail.com Neonatal Intensive Care Unit in SOMER Clinic Rionegro Colombia macalodri12@gmail.com; 4 Nurse, Specialist. Neonatal Intensive Care Unit in SOMER Clinic, Rionegro, ColombiaEmail: manu1152@hotmail.com Neonatal Intensive Care Unit in SOMER Clinic Rionegro Colombia manu1152@hotmail.com; 5 Health Information Systems Manager, Master. Teacher. CES University, Medellín, Colombia. Email: gabrieljlp@gmail.com CES University Medellín Colombia gabrieljlp@gmail.com

**Keywords:** infant, premature, kangaroo-mother care method, breast feeding, health personnel, recién nacido prematuro, método madre-canguro, lactancia materna, personal de salud., recém-nascido prematuro, método canguru, aleitamento materno, pessoal de saúde.

## Abstract

**Goal.:**

To determine the factors associated with the duration of breastfeeding in mothers of babies cared for in a kangaroo family program.

**Methods.:**

Quantitative, observational study with a secondary source of a retrospective cohort of 707 babies with monitoring at admission, at 40 weeks, at three and at six months of corrected age in the kangaroo family program of a public hospital in the municipality of Rionegro (Antioquia, Colombia) from 2016 to 2019.

**Results.:**

49.6% of babies were born with low weight for gestational age and 51.5% were female. 58.3% of the mothers were unemployed and 86.2% of them lived with their partner. When entering the kangaroo family program, 94.2% of the babies received breastfeeding and at six months they were 44.7%. The variables that were associated with the duration of breastfeeding up to six months according to the explanatory model were: the mother's cohabitation with her partner (adjusted prevalence ratio - APR: 1.34) and receiving breastfeeding when entering the kangaroo family program (APR: 2.30).

**Conclusion.:**

The factors related to the duration of breastfeeding in mothers of babies cared for in the kangaroo family program were that the mother lived with her partner and that the mother was breastfeeding when she entered the program, therefore they received education and support from the interdisciplinary team, which could favor confidence and willingness towards breastfeeding.

## Resumen

**Objetivo.:**

Determinar los factores asociados a la duración de la lactancia en madres de bebés atendidos en un programa de familia canguro.

**Métodos.:**

Estudio cuantitativo, observacional con fuente secundaria de una cohorte retrospectiva de 707 bebés con seguimiento al ingreso, a las 40 semanas, a los tres y a los seis meses de edad corregida en el programa familia canguro de un hospital público del municipio de Rionegro (Antioquia, Colombia) entre 2016 y 2019.

**Resultados.:**

El 49.6 % de los bebés nacieron con bajo peso para la edad gestacional y 51.5% eran de sexo femenino. El 58.3% de las madres eran desempleadas y un 86.2 % de ellas convivía con su pareja. Al ingresar al programa familia canguro, el 94.2 % de los bebés recibían lactancia materna y a los seis meses correspondió al 44.7%. Las variables que se asociaron con la duración de la lactancia materna hasta los seis meses según el modelo explicativo fueron: la convivencia de la madre con la pareja (razón de prevalencia ajustada - RPa: 1.34) y recibir lactancia al ingresar al programa familia canguro (RPa: 2.30).

**Conclusión.:**

Los factores que se relacionaron con la duración de la lactancia en madres de bebés atendidos en el programa de familia canguro fueron, por un lado, que la madre conviviera con su pareja y, por otro, que la madre estuviera lactando al ingreso al programa, por lo que recibieron educación y acompañamiento por parte del equipo interdisciplinario, lo cual pudo favorecer la confianza y disposición hacia la lactancia.

## Resumo

**Objetivo.:**

Determinar os fatores associados à duração do aleitamento materno em mães de bebês atendidos em um programa família canguru.

**Métodos.:**

Estudo quantitativo, observacional com fonte secundária de uma coorte retrospectiva de 707 bebês com acompanhamento na admissão, às 40 semanas, aos três e seis meses de idade corrigida no programa família canguru de um hospital público do município de Rionegro (Antioquia, Colômbia) entre 2016 e 2019.

**Resultados.:**

49.6% dos bebês nasceram com baixo peso para a idade gestacional e 51.5% eram do sexo feminino. 58.3% das mães estavam desempregadas e 86.2% delas moravam com o companheiro. Ao ingressar no programa família canguru, 94.2% dos bebês receberam aleitamento materno e aos seis meses correspondeu a 44.7%. As variáveis que se associaram à duração do aleitamento materno até seis meses segundo o modelo explicativo foram: a coabitação da mãe com o companheiro (razão de prevalência ajustada - TAEG : 1.34) e receber aleitamento materno ao ingressar no programa família canguru (RPa: 2.30) .

**Conclusão.:**

Os fatores que se relacionaram com a duração do aleitamento materno em mães de bebês atendidos no programa família canguru foram que a mãe morava com o companheiro e que a mãe estava amamentando quando ingressou no programa, para o qual receberam educação e apoio da da equipe interdisciplinar, o que poderia favorecer a confiança e disposição para a amamentação.

## Introduction

Complications associated with prematurity and low birth weight are an important cause of morbidity and mortality.^(^1) Therefore, babies born with any of these conditions should receive care under the Kangaroo Mother Care Method (KMC) guidelines, which corresponds to a set of protocolized and standardized care for high-risk newborns that is based on skin-to-skin contact, feeding ideally based on breast milk and a timely hospital discharge, with the purpose of empowering parents in the baby care and detecting early warning signs. Likewise, they require monitoring by the multidisciplinary team of the outpatient program in order to identify developmental alterations and intervene them at the appropriate time.^(^21)

Various investigations support the benefits of KMC for babies, the family, and society highlighting its effect on breastfeeding, which is part of the components of kangaroo care. Feeding a preterm infant has special considerations related to the specific characteristics of milk, concerns about weight gain, and fear associated with breastfeeding.(3) Therefore, the self-efficacy and confidence of preterm infants’ mothers regarding their ability to breastfeed are factors related to the duration and exclusivity of breastfeeding.(4) While breastfeeding rates have now improved among very low birth weight and normal weight infants, figures for those with low birth weight (between 1,500 g and < 2,500 grams) are further off target, likely because no answer is given to the need for support that the mothers of these babies have to breastfeed. This highlights the need to promote strategies aimed at ensuring that all newborns are breastfed.(5) Kangaroo care is one of the predictive factors of a longer duration of breastfeeding in preterm newborns and contributes to its increase up to 4.1 times compared to those who do not receive this care system.(6) Similarly, the early start of skin-to-skin contact between the mother and her child has a positive effect on the duration of breastfeeding up to six months.(7) Aspects such as the father’s positive attitude, his participation, and his support for the mother during this process influence the mother’s decision and commitment to breastfeed for a longer time.^(^8) Educational and employment aspects also have an effect on breastfeeding. Some investigations report a higher proportion and duration of this practice among women with a better educational level,(9) while, among female workers, these indicators are lower mainly when they return to their jobs.(10)

Health workers have a fundamental role related to exclusive and continuous breastfeeding. A study carried out with nurses and mothers of preterm newborns showed that nursing interventions to accompany, guide, and encourage women to practice breastfeeding have a positive effect on their abilities and willingness to breastfeed.(11) Similarly, care focused on premature babies and their families, as well as institutional policies to promote breastfeeding, favor the mothers’ conditions and willingness to practice it.(12) Based on the above, the purpose of this study was to determine the factors associated with the duration of breastfeeding up to six months of corrected age in a group of children cared for in the kangaroo family program of a municipality located in the department of Antioquia. (Colombia).

## Methods

Quantitative, observational, analytical study of a retrospective cohort of 707 children treated in the kangaroo family program of a public hospital in the municipality of Rionegro (Antioquia, Colombia). The inclusion criterion was attendance at the six-month CA (Corrected Age) control. Patients with hypoxic ischemic encephalopathy and grade III and IV intraventricular hemorrhage were excluded. None of the patients had a contraindication for breastfeeding or palatal defects. The corrected age is defined as the chronological age in days or weeks that the baby was missing to complete 40 weeks of gestation.(13) The source of the information corresponded to the database collected by the program staff on the interventions with the children in the different monitoring periods that corresponded to admission, 40 weeks, three and six months of CA. The interdisciplinary team consisted of pediatrics, nursing professionals and assistants, psychology, physiotherapy, speech therapy, optometry, and retinology. Control care included physical examination, developmental assessment, stimulation techniques, evaluation of the socio-affective context, and education on breastfeeding positions, signs of proper breast grip, tube or syringe feeding techniques, re-breastfeeding, baby care at home and alarm signs. In each control, the type of feeding was asked but it was not possible to determine the proportion of babies exclusively breastfed up to six months of CA, since this variable was not included in the database and when they were seen at this appointment a high proportion was already receiving complementary feeding.

The study had the approval of the ethics committee of the health institution and the Catholic University of the East, classifying it as risk-free research in accordance with the Colombian resolution that establishes the scientific, technical, and administrative standards for health research.(14) The identification data of the children and their families were anonymized to preserve their privacy.

The descriptive analysis of the variables was done according to their nature. For the qualitative ones, relative frequencies were calculated; and for the quantitative ones, the average was calculated with its measure of dispersion after evaluating the normality in its distribution with the Kolmogorov Smirnov test. Bivariate analysis was performed to establish the association of the dependent variable (duration of breastfeeding) with each of the selected independent variables using the Chi2 test with a significance level of 5% (p<0.05) and crude RPs were calculated. In the multivariate analysis, all the selected variables were included and through logistic regression with the Enter method, the adjusted ORs were generated. The level of significance was established at 5% (p<0.05). Since the multivariate model generates the OR association measure, the Stromberg (15) formula was used to convert it to RP. The analyzes were performed with the R statistical package.

## Results

The gender distribution was similar and about half of the cohort were classified as term newborns with low weight for gestational age (RNAT-PBEG, abbreviation in Spanish). The GA average was 35.8 weeks (SD: 2.2), birth weight was 2228.7 grams (SD 382.6), and height was 45.7 centimeters (SD 3.1). The mothers’ average age was 27 years old (SD: 6.4) and the fathers’ average age was 31 years old (SD: 7.6). At the six-month CA control, about half of the children were still being breastfed. Characterization data are presented in Table 1.

**Table 1:** Sociodemographic, economic and clinical characteristics of babies cared for in the kangaroo family program

Variable	Category	*n*	%
Pregnancy	Planned	400	56.6
	Unplanned	307	43.4
Type of housing	Rural	244	34.5
	Urban	463	65.5
Social security system	Contributory	497	70.3
	Subsidized	210	29.7
Cohabitation of the mother with the couple	Cohabits	609	86.2
	Does not cohabit	98	13.8
Mother schooling	Primary or high school	450	63.6
	Technique or technology	152	21.5
	Professional or specialist	105	14.9
Mother occupation	Unemployed	412	58.3
	Employed	295	41.7
Works during pregnancy	Yes	335	47.4
	No	372	52.6
Father schooling	Primary or high school	478	67.6
	Technique or technology	98	13.9
	Professional or specialist	80	11.3
	No data	51	7.2
Father occupation	Unemployed	9	1.3
	Employed	655	92.7
	No data	43	6.0
Monthly family income	More than minimum wage	411	58.1
	A minimum wage or less	296	41.9
Family Nutrition (servings)	3 servings or less	289	40.9
	4 servings or more	418	59.1
Sex of the newborn	Female	364	51.5
	Male	343	48.5
Birth weight/gestational age classification	RNAT-PBEG	351	49.6
	RNPT-PAEG	191	27.0
	RNPT-PBEG	165	23.3
Type of birth	Spontaneous vertex birth	400	56.6
	Caesarean section birth	307	43.4
Birth hospitalization	Yes	432	61.1
	No	275	38.9
Breastfeeding upon admission to the KFP	Yes	667	94.3
	No	40	5.6

**Figure 1:**
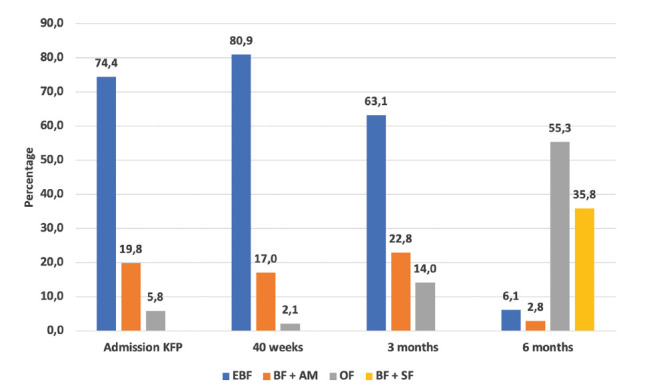
Distribution of children according to the type of feeding in the controls of the kangaroo family program Note: EBF (exclusive breastfeeding); BF + AM (breastfeeding + artificial milk); BF + SF (breastfeeding + supplementary feeding); Other food (artificial milk, supplementary feeding or both).

The variables that were associated with breastfeeding in the unadjusted analyzes were: number of control appointments attended in the kangaroo family program up to six months of corrected age, height at birth, health system, type of housing, cohabitation of the mother with the couple, the mother's occupation, family nutrition, having worked during the pregnancy, type of birth and breastfeeding upon admission to the program. These variables presented a significant association (p<0.05) with the dependent variable. Once the explanatory model was made, adjusting for all the variables that were associated in the bivariate analysis, only the cohabitation of the mother with the couple (APR 1.64; 95% CI: 1.04-2.61) and breastfeeding at the time of admission to the program (APR 3.17; 95% CI: 1.37-8.31) maintained their significant association with breastfeeding at six months. See Table 2.

**Table 2:** Sociodemographic, economic and clinical factors associated with the duration of breastfeeding at six months of corrected age

Variable		Breastfeeding at six months				PR (IC95%)	Chi2	*P*	APR’ (IC95%)	APR’ (IC95%)
		Yes		No						
		n	%	n	%					
Number of control appointments.		3.71± 0.55		3.61±	0.65	1.325 (1.036 - 1.707)	2.27	0.02	1.15 (0.82 - 1.61)	-
Average ± DE										
Height when entering the program		47.13	± 4.09	47.97±	4.94	0.96 (0.92 -0.99)	-24.83	0.01	0.99 (0.94 - 1.03)	-
Average ± DE Type of housing	Urban	198	0.63	265	0.68	0.88 (0.75 - 1.05)	2.02	0.15	1.20 (0.86 - 1.67)	1.09 (0.86-1.67)
	Rural*	118	0.37	126	0.32					
Cohabits with the	Yes	282	0.89	327	0.84	1.33 (1.00 - 1.77)	4.60	0.03	1.64 (1.04 - 2.61)	1.34 (1.04-2.58)
couple	No*	34	0.11	64	0.16					
Mother’s occupa-	Employed	123	0.39	172	0.44	0.89 (0.75 - 1.05)	1.84	0.17	0.94 (0.61 - 1.45)	0.97 (0.61-1.45)
tion	Unemployed*	193	0.61	219	0.56					
Family nutrition	4 servings or	199	0.63	219	0.56	1.18 (0.99 - 1.40)	3.51	0.06	1.26 (0.92 - 1.73)	1.14 (0.92-1.72)
	more									
	3 servings or less*	117	0.37	172	0.44					
Worked during	Yes	140	0.44	195	0.50	0.88 (0.75 - 1.04)	2.17	0.14	0.90 (0.59 - 1.36)	0.94 (0.59-1.35)
pregnancy	No*	176	0.56	196	0.50					
Type of birth	SVB	189	0.60	211	0.54	1.14 (0.96 - 1.35)	2.43	0.12	1.20 (0.89 - 1.64)	1.11 (0.88-1.64)
	Caesarean*	127	0.40	180	0.46					
Breastfeeding	Yes	309	0.98	358	0.91	2.65 (1.34 - 5.21)	12.69	0.00	3.17 (1.37 - 8.31)	2.30 (1.30-7.72)
when entering the program	No*	7	0.02	33	0.08					
Health system	Contributory	217	0.69	289	0.74	0.87 (0.73 - 1.03)	2.36	0.12	0.80 (0.55 - 1.15)	0.88 (0.55-1.15)
	Subsidized*	99	0.31	102	0.26					

*Reference category; APR: adjusted prevalence ratio; APR†: adjusted prevalence ratio with conversion from OR to PR using the Stromberg formula

## Discussion

In this study, which aimed to determine the factors associated with the duration of breastfeeding up to six months in mothers of babies cared for in a kangaroo family program, the findings apply to families that were monitored up to six months of CA and they cannot be widespread to all program participants. Lizarazo-Medina *et al.*(16) carried out an investigation with premature and low birth weight babies to describe the effectiveness and achievements of the kangaroo mother program on their development. They found that when entering the KFP, half of the babies received exclusive breastfeeding and during the control at 40 weeks of corrected age, the proportion increased to 82%. In the present study, the first datum corresponded to about 75% of the babies and the second was similar (80.9%). This increase in the proportion of breastfeeding from admission to the 40-week control in both studies may be related to the education that the nursing staff of the program provides families on the care of the premature baby at home and on the lactation techniques.

In the present investigation it was not possible to establish the proportion of exclusive breastfeeding up to six months of corrected age, since, in a high proportion of cases, the babies’ attendance to this control was later and the families had already incorporated the supplementary feeding. However, it was identified that 63.1% were exclusively breastfed at the three-month control and about half (44.7%) were non-exclusively breastfed at six months of corrected age. Montealegre-Pomar *et al.* (^17^) monitored 1138 premature and low birth weight infants in the Yopal kangaroo mother program and they determined that exclusive breastfeeding at six months was 59.2%. These data allow a comparison to be made with the results of the national nutritional status survey,(18) which reports a proportion of exclusive breastfeeding of 36.1% in children under six months of age, a lower percentage than the one found in both investigations. One possible explanation for why the prevalence of exclusive breastfeeding in children under six months in Colombia is lower than in kangaroo babies is the positive effect that monitoring by the interdisciplinary team of the program has on the decision and commitment of families for guaranteeing a longer duration of breastfeeding in this population which requires a special care system.

One of the fundamental axes of the kangaroo mother method is the feeding of the premature or low birth weight newborn, ideally with breast milk.(^19^) Therefore, identifying the factors associated with the duration of breastfeeding in this specific population contributes to promoting them in order to improve the results of kangaroo care. Encouraging mothers to practice kangaroo care and ensuring the appropriate conditions for it has a protective effect on breastfeeding in preterm and/or low birth weight newborns,(20,21) therefore, the support they receive from their family, and in particular from their partners, is a factor that favors this practice. In the present investigation, it was found that the cohabitation of the mother with her partner is an explanatory factor for breastfeeding the kangaroo baby up to six months of corrected age. Chugh *et al*.(22) report similar findings, since it is more difficult to continue exclusive breastfeeding for women who do not receive support from their family members in household chores, while the fathers’ support favors this practice. Likewise, it was found in another study that the formation of a team between the woman and her partner favors kangaroo care and breastfeeding according to they take turns on the responsibilities so that the mother can rest or express milk while the baby is in skin-to-skin contact with the father.(23)

Timely hospital discharge with the guarantee of constant monitoring is a component of the KMC, from which emotional, physical and educational support is provided to the mother and family to favor the satisfaction of the kangaroo baby needs.(24) The application of the method can start from birth when skin-to-skin contact is established between the mother and the baby, which has a beneficial effect on breastfeeding.(25) This implies that the KMC starts from the hospital and it must be continued after discharge. In the present study, it was found that breastfeeding at admission to the KFP is an explanatory factor for the fact that kangaroo babies were breastfed up to six months of corrected age. The orientations that mothers receive from the health personnel during the kangaroo adaptation in the hospital, the education in maternal feeding techniques of the premature baby, and the monitoring by the interdisciplinary team of the kangaroo family program are factors that contribute to improving the confidence of mothers in their ability to breastfeed their premature child, which is evidenced as a factor that influences the duration of breastfeeding.(26) As stated by Sinha *et al.*,(27) advice to mothers by the health team contributes to improving the rates of early initiation, exclusivity and the total duration of breastfeeding. 

The nursing staff plays an important role in the hospital and outpatient kangaroo care team, since they are responsible for adapting the premature baby to the KMC, monitoring their anthropometric parameters, guiding the mother and evaluating the quality of breastfeeding, and support the family in preparing for the baby care at home. Therefore, it is important that the interventions of the KFP interdisciplinary team contribute to the goal of improving breastfeeding rates in this population.

The conclusion of this study is that among the sociodemographic, economic, and clinical factors evaluated, the cohabitation of the premature babies’ mothers with their partner and breastfeeding upon admission to the kangaroo family program were factors that were associated with the duration of breastfeeding in the explanatory model. Mothers who live with their partner have the possibility of sharing the responsibility of caring for the baby, and those who offer breastfeeding upon entering the program receive education and support from the interdisciplinary team, all of which can promote their confidence and willingness to breastfeed.

It is necessary to identify more adjustable factors that influence the duration of breastfeeding in kangaroo babies that can be the object of interventions, as well as it must be documented which specific interventions that are carried out in the KFP are more useful to contribute to this purpose.

One of the limitations of this study is that it was not possible to establish the prevalence of exclusive breastfeeding up to six months of corrected age because at the time of the study this variable was not defined in the database, since retrospective data were used which were collected with clinical and non-investigative purposes. Therefore, the database did not contain information on the rate of loss to monitoring in the program and on a greater number of economic, social, educational, and work variables that can influence the duration of breastfeeding. Another limitation is that most of the explored variables correspond to sociodemographic or birth factors that are not adjustable.

Grant. This article is derived from a research project entitled "Demographic, social, economic, and clinical factors that influence the duration of breastfeeding in high-risk patients who were monitored in the kangaroo family program in the municipality of Rionegro, between 12/16/2016 and 12/31/2019”, which was financed by the Directorate of Research, Development and Innovation of the Catholic University of the East.
